# How Bacteria Turn Fiber into Food

**DOI:** 10.1371/journal.pbio.1001227

**Published:** 2011-12-20

**Authors:** Mason Inman

**Affiliations:** Freelance Science Writer, Berkeley, California, United States of America

**Figure pbio-1001227-g001:**
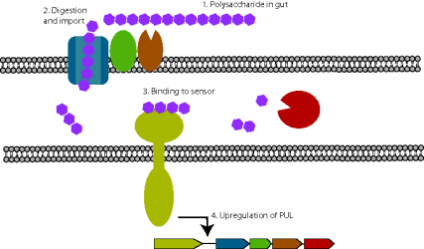
Highly specific membrane protein complexes enable gut bacteria to sense and degrade specific plant cell wall polysaccharides and augment human digestive capabilities.

[Fig pbio-1001227-g001]Most people think of fiber as being the part of our food we can't digest—but with the help of symbiotic bacteria in our guts, we can actually get some nutrition from the complex carbohydrates such as cellulose, hemicellulose, and pectin that make up plants' cell walls. The bacteria produce an arsenal of enzymes that break down these carbs into simple sugars, which are then in turn fermented to create short-chain fatty acids that human cells can absorb—and which can contribute as much as 10 percent of the calories our own cells require.

These gut bacteria are even more adept at breaking down complex carbohydrates than had been previously thought, according to a new study in *PLoS Biology* that sheds new light on how these bacteria are able to play this crucial role.

Recent studies have found that the bacteria use complexes of proteins that span the bacterial outer membrane and allow them to capture and break down complex polysaccharides into their monosaccharide components. Sets of genes known as polysaccharide utilization loci (PULs) each encode a toolkit of proteins needed to recognize, import, and digest a specific polysaccharide or set of related polysaccharides; each PUL also contains genes encoding a sensor-regulator that can relay information about what is happening outside the cell. But exactly how the bacteria use these protein complexes and sensors in their daily life has been unclear.

Now, a new study by Eric Martens, David Bolam, and colleagues has looked into how a pair of the most common species of gut bacteria metabolize polysaccharides, showing that each bacterium is highly specialized. Using a high-throughput system for feeding the bacteria dozens of kinds of carbohydrates, one at a time, and tracking the bacteria's gene expression, they were able to see how these microbes have tailored themselves to fill specific niches in the gut.

These two bacterial species do have a fair amount of overlap in what they are able to subsist on, including the ability to eat more complex carbohydrates than had been thought possible—with PULs encoding enzymes that break down even rhamnogalacturonan II, a very complex carbohydrate that's a common part of plant cell walls, and that had been thought to be resistant to microbes.

But the two species—both from *Bacteroides*, one of the main genera of gut bacteria in mammals—have diverged to tackle particular kinds of foods. *Bacteroides ovatus* can break down hemicelluloses. *Bacteroides thetaiotaomicron*, though, can't eat these hemicelluloses—but it does have a suite of PULs that help it break down many pectins, as well as other PULs that target complex carbohydrates that human intestinal cells secrete as mucus.

The new results also help explain a counterintuitive result from earlier work, which found that *B. thetaiotaomicron* grew faster on polysaccharides than it did on the monosaccharides that made up those polysaccharides. This was surprising, since to metabolize the polysaccharides, the bacteria first have to break them down into sugars.

To figure out the role the sensor-regulators might play in this, Martens, Bolam, and colleagues isolated those portions of four different sensor-regulator proteins that are exposed on the bacteria's cell membrane, and so should be able to interact directly with fragments of polysaccharide generated by the PUL-encoded enzymes. Their measurements of how strongly these proteins bound to various fragments of polysaccharide reveal these sensor-regulator proteins are able to detect particular bonds between sugars that are characteristic of each polysaccharide.

Because this recognition is so specific, the sensors potentially enable the bacteria to recognize when a particular polysaccharide is present, and to activate only those PULs that encode the right enzymes for breaking down that complex carbohydrate. This specificity also explains the surprising results of earlier work, in which the bacteria didn't grow as well on simple sugars as on polysaccharides. It seems the bacteria's sensors are tuned mainly toward complex carbohydrates and that the bacteria are largely blind to many simple sugars, even when they're literally swimming in them. While they could in principle survive on these sugars, they have evolved to ignore them, most likely because they are not present in their environment, as the host organism absorbs these simple molecules early on in the digestive tract.

So despite the close evolutionary relationship between *B. thetaiotaomicron* and *B. ovatus*—they share ribosomal DNA that is more than 96% identical—it appears, the new study argues, that they have diverged to fill specific niches, focusing on particular types of carbohydrates available in the gut.

The PULs are spread throughout the bacteria's genome, rather than being grouped in one bunch. The authors suggest that this supports the idea that the bugs largely picked up these traits piece-wise, possibly via horizontal transfer and natural selection.

Microbes are now being engineered to break down plant cell wall polysaccharides to turn them into biofuels, and a better understanding of how gut bacteria metabolize these molecules could aid such industrial applications. Closer to home, these bacteria are also a crucial part of the “superorganism” of human and bacterial cells that make up our bodies, so further understanding the gut bacteria's metabolism could also help heal the sick or improve nutrition.

**Martens EC, Lowe EC, Chiang H, Pudlo NA, Wu M, et al. (2011) Recognition and Degradation of Plant Cell Wall Polysaccharides by Two Human Gut Symbionts. doi:10.1371/journal.pbio.1001221**

